# Ipsilateral foetal-type posterior cerebral artery is associated with cognitive decline after carotid revascularisation

**DOI:** 10.1186/1471-2377-14-84

**Published:** 2014-04-16

**Authors:** Aysun Altinbas, Jeroen Hendrikse, Ale Algra, Martine JE van Zandvoort, Martin M Brown, Leo H Bonati, Gert Jan de Borst, L Jaap Kappelle, H Bart van der Worp

**Affiliations:** 1 Utrecht Stroke Center, Departments of Neurology, Rudolf Magnus Institute of Neuroscience, University Medical Center Utrecht, Utrecht, The Netherlands; 2 Utrecht Stroke Center, Departments of Radiology, Rudolf Magnus Institute of Neuroscience, University Medical Center Utrecht, Utrecht, The Netherlands; 3 Julius Center for Health Sciences and Primary Care, University Medical Center Utrecht, Utrecht, The Netherlands; 4 Department of Experimental Psychology, Helmholtz Institute, Utrecht University, Utrecht, The Netherlands; 5 Department of Brain Repair and Rehabilitation, Institute of Neurology, University College London, London, UK; 6 Department of Neurology and Stroke Unit, University Hospital Basel, Basel, Switzerland; 7 Vascular Surgery, University Medical Center Utrecht, Utrecht, The Netherlands; 8 Department of Neurology, Rudolf Magnus Institute of Neuroscience, University Medical Center Utrecht, P.O. Box 85500, G03.228, 3508 GA Utrecht, The Netherlands

**Keywords:** Angioplasty and stenting, Carotid endarterectomy, Symptomatic carotid stenosis, Cognition, Circle of Willis

## Abstract

**Background:**

Stenosis of the internal carotid artery has been associated with cognitive impairment and decline. However, studies testing the effect of carotid revascularisation on cognition have had conflicting results. This may in part be explained by variation in the flow territory of the carotid artery. In 12 to 36% of the patients, the posterior cerebral artery is mainly or exclusively supplied by the internal carotid artery via a foetal-type posterior cerebral artery. In these patients, ipsilateral carotid artery stenosis is likely to result in a larger area with hypoperfusion than in case of a normal posterior cerebral artery. Patients with a foetal-type posterior cerebral artery could therefore benefit more from revascularisation. We compared the effects of carotid revascularisation on cognition between patients with a foetal-type and those with a normal posterior cerebral artery.

**Methods:**

Patients with symptomatic internal carotid artery stenosis ≥ 50%, enrolled in the International Carotid Stenting Study (ICSS) at a single centre, underwent detailed neuropsychological examinations before and 6 months after revascularisation. Cognitive test results were standardized into z-scores, from which a cognitive sumscore was calculated. The primary outcome was the change in cognitive sumscore between baseline and follow-up. Changes in cognitive sumscore were compared between patients with an ipsilateral foetal-type and those with a normal posterior cerebral artery, as assessed with CT or MR angiography.

**Results:**

Of 145 patients enrolled in ICSS at the centre during the study period, 98 had both angiography at baseline and neuropsychological examination at baseline and at 6-months follow-up. The cognitive sum score decreased by 0.28 (95% confidence interval, 0.10 to 0.45) in 13 patients with an ipsilateral foetal-type posterior cerebral artery and by 0.07 (95% CI, 0.002 to 0.15) in 85 patients with a normal posterior cerebral artery (mean difference, -0.20; 95% CI, -0.40 to -0.01). This did not change essentially after adjustment for baseline factors.

**Conclusion:**

An ipsilateral foetal-type posterior cerebral artery appears to increase cognitive decline after carotid revascularisation. Our findings have to be reproduced in an independent study before further implications can be made.

## Background

In population-based studies, internal carotid artery (ICA) stenosis has been associated with cognitive impairment and decline [1,2]. Although convincing evidence supporting a causal relationship is lacking, this has been attributed to cerebral hypoperfusion or to the occurrence of ‘silent’ brain infarcts [3]. In patients with symptomatic ICA stenosis, perfusion of the ipsilateral territory of the middle cerebral artery is inversely related to the degree of the stenosis [4]. Several authors have therefore suggested that in these patients, carotid revascularisation could improve cognition. However, studies assessing the effect of carotid revascularisation on cognition have not been able to demonstrate a convincing benefit [5]. A recent substudy of the randomized International Carotid Stenting Study (ICSS) [6] even found that carotid artery stenting (CAS) was associated with a small decrease in cognition at 6 months after the procedure. In this study, carotid endarterectomy (CEA) had no effect on cognition [7].

The conflicting results of studies testing the relation between carotid revascularisation and changes in cognition have been ascribed to differences between the studies in sample size, type of patients, duration of follow-up, and type of neuropsychological assessment [5]. Differences in effect of revascularisation on cognition might also be explained by variation in the territory of the brain supplied by the carotid artery between individual patients. Considerable variation of the circle of Willis has been described in healthy individuals [8], as well as in patients with symptomatic ICA stenosis [9]. A foetal-type configuration of the posterior part of the circle of Willis (FTP) has been found in 12 to 36% of the cases. In these cases, the posterior cerebral artery is mainly or exclusively supplied by the ICA via the posterior communicating artery (PCoA) [10]. In patients with a foetal-type PCoA, an ipsilateral ICA stenosis may result in a larger volume with hypoperfusion than in patients with a normal variant of the circle of Willis. If cerebral perfusion is related to cognition, this may result in more severe cognitive impairment. Alternatively, emboli from ipsilateral ICA stenosis might cause damage in areas supplied by a foetal-type PCoA, contributing to cognitive dysfunction.

The (infero)medial temporal lobe and occipital two thirds of the hippocampus are commonly supplied by posterior cerebral artery (PCA) branches [11], and are therefore dependent on the ICA in case of a foetal-type PCoA. This part of the hippocampus is critical for cognition, especially for memory and spatial navigation [12]. A recent study assessing cognitive function during intracarotid amobarbital infusion in epilepsy patients found that the presence of an FTP was associated with lower memory scores [13].

We hypothesized that the benefit of carotid revascularisation with regard to cognition would be larger in patients with an ipsilateral foetal-type circle of Willis than in patients with the normal variant. Therefore, we compared changes in cognition 6 months after CEA or CAS for symptomatic ICA stenosis between patients with a foetal-type FTP and those with a normal PCA.

## Methods

The present study is a single-centre prospective substudy of the randomized International Carotid Stenting Study (ICSS) [6]. All patients enrolled in ICSS at the University Medical Center Utrecht, the Netherlands, between February 2006 and December 2008, were considered for inclusion in this substudy. ICSS was an international, randomized, controlled, clinical trial comparing CAS and CEA in patients with a recently symptomatic carotid artery stenosis ≥ 50%. Centre and investigator requirements for participating in ICSS, patient eligibility criteria, study procedures, clinical follow-up examinations, and an interim safety analysis have been described elsewhere [6]. We have earlier reported the effects on cognition of CAS and CEA in this substudy of ICSS [7]. Patients were excluded from the present study in case of pre-existent cognitive decline, a language barrier, when a neuropsychological evaluation was not done or not informative because of severe aphasia or other severe focal neurological deficit, or when the intracranial arteries were not visualized with CT or MR angiography.

### Study approval

The Medical Research Ethics Committee UMCU approved both ICSS (ISRCTN25337470) and the cognitive substudy, and each patient provided written informed consent.

### Study sample and assessments

Patients with recently symptomatic ICA stenosis of at least 50%, measured according to the North American Symptomatic Carotid Endarterectomy Trial criteria or its non-invasive equivalent [14], were randomly assigned to CAS or CEA. At inclusion, data were collected on presenting symptoms, demographic characteristics, and cardiovascular risk factors. The score on the National Institutes of Health Stroke Scale (NIHSS) was assessed at baseline, and the score on the modified Rankin Scale (mRS) at baseline and at 1 and 6 months after the procedure.

### Neuropsychological assessment and test transformation

Cognition was assessed in the week preceding the procedure and after 6 months. The neuropsychological examination (NPE) comprised 7 cognitive domains, including 15 tasks, as described previously [7]. In addition, mood was assessed by the Beck Depression Inventory II [15], and anxiety by the Spielberger State and Trait Anxiety Inventory [16]. Premorbid verbal intelligence was estimated with the National Adult Reading Test (NART, Dutch version) [17], pre-existent cognitive decline was defined as a score of 3.6 or higher on the Informant Questionnaire of Cognitive Decline in the Elderly [18], and current general cognitive functioning with the Mini Mental State Examination [19].

Individual raw test scores at baseline and at follow-up were expressed as standard deviation (SD) units; the so-called z-scores. Z-score transformation was based on the mean and SD of a historical control group [20], in which healthy individuals performed the same NPE at similar time intervals, thus controlling for potential practice effects in our patients. The control group consisted of 75 persons who were matched to acute stroke patients with a mean age of 60 years. Each z-domain score represents the mean of the z-scores in that domain. As a measure of overall cognitive functioning, a cognitive sumscore was calculated, representing the mean z score over the 7 domains [21]. NPEs were rated by a single observer (Ay.A) and were screened for accuracy by an experienced clinical neuropsychologist (MJEvZ). Both were blinded to the anatomy of the circle of Willis in the individual patients.

### Methods

#### Imaging parameters

CT angiography (CTA) or MR angiography (MRA) was performed in the week before revascularization (Additional file 1).

All CT- and MR-angiograms were evaluated by a neuroradiologist (JH) who was blinded to treatment assignment and to clinical and cognitive outcomes. In each patient, all segments of the circle of Willis were evaluated: anterior communicating artery (ACoA, present or absent); A1 segment of the anterior cerebral artery (ACA; normal, hypoplastic, or absent), P1 segment of the PCA (present or absent), and the PCoA (absent, or diameter < P1, =P1, or > P1) [9]. Foetal-type PCA (FTP) was defined as a PCoA with a diameter greater than that of the ipsilateral P1, or when the ipsilateral P1 was absent [22].

#### Diffusion imaging

MRI with DWI was performed 1 to 3 days before revascularisation and within 3 days thereafter. The number and volume of new ischaemic lesions were assessed as part of the previously reported ICSS-MRI study [23]. A new ischaemic lesion was defined as a new hyperintensity on the post-treatment DWI that was not present on the pretreatment MRI. Lesions were silent if there was no new corresponding focal neurologic deficit.

#### Outcome measures

The primary outcome measure of this study was the change in cognitive sum z-score between baseline and follow-up. A decrease in z-score means a worsening of cognition from baseline to follow-up. Secondary outcome measures were the changes in individual cognitive domain scores.

#### Statistical analysis

We performed t-tests for continuous and normally distributed data, Mann-Whitney U tests for non-parametric data, and χ^2^ analyses for categorical data on baseline characteristics to determine whether selection bias had occurred between patients who were re-examined at follow-up and those who were not. Because of the sample size we could not perform reliable subgroup analyses based on the treatment received.

We calculated the mean difference in the change of the cognitive sum z-score between baseline and follow-up between the patients with and without FTP with corresponding 95% confidence intervals (CIs) with linear regression. Adjusted mean differences were calculated and are reported for those factors that changed the point estimate with at least 10%. Similar calculations were done for domain scores.

## Results

### Patient flow and baseline characteristics

During the study period, 145 patients were randomized in ICSS at our centre. Five of these patients had no revascularisation because of medical reasons (severe disability (3) or carotid occlusion (2)), four had pre-existent cognitive decline, four refused to participate, one had a language barrier, one had an unreliable baseline NPE, 10 had no cranial CTA or MRA, and 8 had logistical reasons for exclusion. Consequently, this study consists of 112 patients with both baseline NPE and angiography, of whom 54 were treated with CEA and 58 with CAS. Sixteen patients had an ipsilateral FTP and 96 patients a normal ipsilateral PCA. Ten patients (63%) with an FTP were treated by CEA and 6 (38%) by CAS. Baseline clinical characteristics and patency of the anterior part of the circle of Willis did not differ between the two groups (Table 1).

**Table 1:** Baseline characteristics

	**Normal PCA**	**Foetal-type PCA**
	**(n = 96)**	**(n = 16)**
Age (years)	69 (9)	68 (9)
Sex (male)	72 (75%)	10 (63%)
Education*	5.0 [4.0–6.0]	4.0 [3.3–4.8]
Vascular risk factors		
Treated hypertension	71 (75%)	10 (65%)
Systolic blood pressure (mm Hg)	168 (27)	174 (28)
Diastolic blood pressure (mm Hg)	86 (13)	87 (13)
Cardiac failure	3 (3%)	1 (6%)
Angina pectoris in past 6 months	7 (7%)	0 (0%)
Previous myocardial infarction	19 (20%)	3 (19%)
Previous CABG	17 (18%)	2 (13%)
Atrial fibrillation	4 (4%)	0 (0%)
Other cardiac embolic source	4 (4%)	0 (0%)
Type 2 diabetes mellitus	16 (17%)	2 (13%)
Type 1 diabetes mellitus	4 (4%)	1 (6%)
Peripheral arterial disease	17 (18%)	3 (19%)
Current smoker	32 (34%)	3 (19%)
Ex-smoker	55 (59%)	9 (56%)
Treated hyperlipidaemia	72 (76%)	13 (81%)
Cholesterol (mmol/L)	4.7 (1.1)	4.9 (1.4)
Imaging		
MRA	68 (71%)	11 (69%)
CTA	28 (29%)	5 (31%)
Treatment		
CEA	44 (46%)	10 (63%)
CAS	52 (54%)	6 (38%)
Symptomatic side		
Left carotid artery	44 (46%)	10 (63%)
Degree of symptomatic carotid artery stenosis†		
50–69%	13 (14%)	2 (13%)
70–99%	83 (87%)	14 (88%)
Degree of contralateral carotid artery stenosis^†^		
<50%	58 (60%)	13 (81%)
50–69%	12 (13%)	0 (0%)
70–99%	15 (16%)	2 (13%)
Occluded	7 (7%)	1 (6%)
Unknown	4 (4%)	0 (0%)
Anatomy anterior part circle of Willis		
Ipsilateral A1 present	80 (83%)	15 (94%)
Anterior communicating artery present	95 (99%)	15 (94%)
Contralateral A1 present	86 (90%)	15 (94%)
Most recent ipsilateral event^‡^		
Amaurosis fugax	24 (26%)	2 (13%)
Transient ischaemic attack	34 (37%)	4 (25%)
Ischaemic stroke	29 (32%)	9 (56%)
Retinal infarction	3 (3%)	1 (6%)
Unknown	2 (2%)	0 (0%)
NIHSS at randomization	0 (0–1)	0.5(0–1)
Modified Rankin score at randomization		
0–2	85 (90%)	15 (94%)
3–5§	10 (11%)	1 (6%)
Unknown	1 (0%)	0 (0%)

Data are number (%), mean (SD), or median (IQR). CABG indicates coronary artery bypass grafting; CAS, carotid artery stenting; CEA, carotid endarterectomy; CTA, computed tomography angiography; MRA; magnetic resonance angiography; NIHSS, national institutes of health stroke scale. *Education levels according to Verhage [24]. ^†^Degree of stenosis measured by NASCET method [2]. ^‡^If two events were reported on the same day, the more serious was counted (stroke > retinal infarction > transient ischaemic attack > amaurosis fugax). ^§^Some Rankin scores of 3 or more were caused by non-stroke disability.

Ninety-eight patients (88%) had a follow-up NPE at six months. Missing follow-up examinations were due to patient refusal (N = 8), severe disability (N = 2), and death (N = 4). Of these patients, three had an ipsilateral FTP. Patients without follow-up NPE were 5.9 years older (95% CI, 0.9 to 10.9), more often had treated hyperlipidemia at baseline (relative risk (RR), 1.35; 95% CI, 1.20 to 1.52), and performed worse on the domain ‘visual memory’ (mean difference (MD), -0.55; 95% CI, -1.09 to -0.01). They did not differ with regard to other demographic, clinical, or baseline cognitive characteristics.

### Cognition

There were no major differences in cognitive sum scores at baseline between the two groups, but patients with an FTP tended to perform worse in each of the cognitive domains and had lower scores for two tasks in the cognitive domains ‘executive functioning’ and ‘visual perception’ (Additional file 1: Table S1).

There was a significant decrease in the cognitive sumscore of 0.28 (95% CI, 0.10 to 0.45) in patients with an FTP from baseline to 6 months follow-up, and of 0.07 (95% CI, 0.002 to 0.15) in patients with a normal variant. The mean difference in sumscore change between patients with and patients without FTP was -0.20 (95% CI, -0.40 to -0.01). After adjustment for potential confounders the results remained essentially the same (Additional file 1: Table S2). Within the individual cognitive domains, there were no statistically significant differences in change from baseline to 6-months follow-up between patients with an FTP and patients with a normal PCA, both before and after adjustment. However, patients with an FTP tended to perform worse in 6 of the 7 domains (Table 2).

**Table 2:** Change in cognitive functioning at 6-months follow-up

**Domain (n normal PCA, n foetal-type PCA)**	**Normal PCA (95% CI)**	**Foetal-type PCA (95% CI)**	**MD (95% CI)**	**MD (95% CI)**
			**Unadjusted**	Adjusted*
Cognitive sum z-score (85,13)	-0.07	-0.28	-0.20	-0.19
	(-0.15 to -0.002)	(-0.45 to -0.10)	(-0.40 to -0.01)	(-0.38 to 0.01)
Cognitive domain z-scores				
Abstract Reasoning (57,9)	-0.05	0.01	0.06	0.07
	(-0.18 to 0.09)	(-0.29 to 0.31)	(-0.30 to 0.41)	(-0.28 to 0.42)
Attention (83,12)	-0.14	-0.72	-0.58	-0.66
	(-0.44 to 0.17)	(-1.32 to -0.11)	(-1.40 to 0.24)	(-1.33 to 0.00)
Executive Functioning (79,12)	0.06	0.05	-0.01	-0.14
	(-0.06 to 0.18)	(-0.35 to 0.45)	(-0.35 to 0.33)	(-0.56 to 0.27)
Language (85,13)	-0.20	-0.24	-0.03	0.13
	(-0.35 to -0.06)	(-0.55 to 0.08)	(-0.42 to 0.36)	(-0.28 to 0.54)
Verbal Memory (84,12)	-0.21	-0.47	-0.26	-0.12
	(-0.36 to -0.06)	(-0.88 to -0.05)	(-0.68 to 0.17)	(-0.60 to 0.37)
Visual Memory (78,12)	0.22	0.11	-0.11	-0.05
	(0.06 to 0.38)	(-0.38 to 0.61)	(-0.54 to 0.33)	(-0.56 to 0.47)
Visual Perception (83,12)	-0.20	-0.21	-0.01	-0.05
	(-0.33 to -0.06)	(-0.62 to 0.20)	(-0.40 to 0.37)	(-0.47 to 0.37)

*Values reflect scores adjusted for side stenosis, time interval between symptom onset and treatment, BDI (Beck’s depression inventory) and STAI, state-trait anxiety inventory.

CI indicates confidence interval; MD, mean difference; PCA, posterior cerebral artery.

The adjusted scores are calculated on fewer patients than described in the first column due to missing values.

### Clinical and DWI outcomes

Fifty-four patients (55%) with follow-up NPE had an MRI within 3 days after the procedure. New ischaemic lesions were found in 17 (35%) of 48 patients with a normal PCA and in 2 (33%) of 6 with an FTP (RR, 0.9; 95% CI, 0.3 to 3.1). Of these lesions, 15 (88%) and 1 (50%) respectively were not associated with stroke or a transient ischaemic attack. There were no differences between the two groups in the median scores on the mRS at one and six months.

2 patients with a normal PCA and 3 patients with an FTP had a stroke within 30 days after the intervention. Excluding these patients from the analyses had no relevant effect on the changes in cognitive sum score and individual domain scores between baseline and 6-months follow-up.

## Discussion

In contrast to our expectations, we found that in patients with a symptomatic ICA stenosis and a foetal-type posterior cerebral artery (FTP), carotid revascularisation was associated with a larger decline in cognition than in patients with a normal PCA. This finding could not be explained by differences in the anterior part of the circle of Willis, by a worse functional outcome at six months after revascularisation, or by a more frequent occurrence of new ‘silent’ ischaemic lesions on DWI within the first 3 days in the subset of patients who had follow-up MRI.

The worse performance in two tasks in the FTP group compared with the PCA group did not result in a greater difference at follow-up. Because we looked at changes in cognition between baseline and follow-up, for this reason any difference in cognition between the two groups at baseline is already accounted for.

In patients with an FTP, the ipsilateral postcommunicating part of the PCA is supplied in whole or in part by the ICA. In these cases, the supply territory of the ICA therefore also includes the posterior part of the thalamus, the posterior two thirds of the hippocampus, the medial-inferior part of the temporal lobe, and the occipital lobe [25]. In patients with an ICA stenosis and an ipsilateral FTP, cerebral perfusion is therefore likely to be compromised in a considerably larger area of the brain than in patients with a normal PCA.

In patients with carotid artery stenosis, perfusion in the territory of the middle cerebral artery is inversely related to the degree of the stenosis [4], and carotid revascularisation has been shown to improve perfusion [26]. Small studies have suggested that an increase in cerebral perfusion after carotid revascularisation is associated with cognitive improvement [27,28]. Because of the larger perfusion territory of the ICA in patients with an ipsilateral FTP, we had hypothesized that any improvement in cognition after carotid revascularisation would be larger in patients with an FTP than in patients with a normal PCA. Our observation of a larger decline in cognition in patients with an FTP is therefore difficult to explain. We found no evidence of a higher rate of new ischaemic brain lesions in patients with an FTP, and there were no differences between the groups in patency of the anterior part of the circle of Willis. We did not find a difference of the point estimate when taking type of treatment into account. However, it is possible that a larger study would find differences in new ischaemic brain lesions and cognitive change between stenting and endarterectomy.

The relation between cerebral perfusion and cognition has remained controversial. In several studies, a lower total cerebral blood flow has been related with worse information-processing speed, executive function, and global cognition [29-31]. However, in one of these studies, these associations disappeared after correcting total cerebral blood flow for brain volume [31]. Still, even if there is no relation between cerebral perfusion and cognition, this would not explain the larger decrease in cognition after revascularisation in patients with an FTP than in those with a normal PCA. Some other studies in patients with ICA occlusion or asymptomatic ICA stenosis have found an association between haemodynamic impairment and cognitive dysfunction [32-34]. Finally, all patients underwent the same detailed neuropsychological examinations, with a global cognitive sumscore as the primary outcome. A recent study has suggested that the use of a cognitive battery that emphasizes the hemisphere ipsilateral to the stenosis may be more sensitive to detect cognitive impairment [34].

The present study has limitations. The small number of patients with an FTP was in line with rates of FTP reported in the literature, but this may have led to imprecision of effect estimates. Although our results did not change essentially after adjustment for potential confounders, we cannot exclude that baseline and postoperative heterogeneity between the two groups may have affected our findings and have resulted in a type I (false-positive) error*.* Secondly, only about half of patients had postprocedural imaging, for which reason we could not reliably assess the effects of number and location of new lesions on cognitive function. Thirdly, we did not assess differences in perfusion of the ipsilateral hemisphere between patients with an FTP and patients with a normal PCA, and do therefore not know whether perfusion in patients with an FTP was indeed more compromised before revascularisation, nor whether this improved more after the procedure. Fourthly, the small number of patients with an FTP did not allow reliable subgroup analyses based on the type of treatment received. However, our results did not change essentially after adjustment for potential confounders, including the method of revascularization. In addition, any difference in cognition at six months after CAS or CEA is likely to be small [7]. Finally, we did not make comparisons of left versus right-sided carotid stenosis and cognitive task performance. Cognitive performance in lateralised tasks and their relation with impaired cerebral reactivity has been identified recently [33].

## Conclusions

In conclusion, an ipsilateral FTP appears to be associated with cognitive decline after carotid revascularisation. The cause of this decline is uncertain, and seems not to be related to the occurrence of periprocedural cerebral ischaemia. Our findings have to be reproduced in an independent study before further implications can be made.

## Competing interests

The authors declare that they have no competing interest.

## Authors’ contributions

Conception and design: AyA, JH, MMB, LJK, HBvdW. Acquisition of data: AyA, JH, MMB, LHB. Analysis and interpretation of data: AyA, JH, AlA, LJK and HbvdW. Drafting of manuscript: AyA and HBvdW. Critically revising the article: AyA, JH, AlA, MJEvZ, MMB, LHB, GJdB, LJK, HBvdW. All authors read and approved the final manuscript.

## Pre-publication history

The pre-publication history for this paper can be accessed here:

http://www.biomedcentral.com/1471-2377/14/84/prepub

## Supplementary Material

Additional file 1 Additional Methods. Click here for file
