# Supplementary material for: Ipsilateral foetal-type posterior cerebral artery is associated with cognitive decline after carotid revascularisation
Source: BMC Neurol. 2014 Apr 16;14:84. doi: 10.1186/1471-2377-14-84 (PMC4021499; doi:10.1186/1471-2377-14-84)
Supplement: Additional file 1 — Additional Methods. [file 1471-2377-14-84-S1.docx]

**Ipsilateral foetal-type posterior cerebral artery is associated with cognitive decline after carotid revascularisation**

**Additional file 1**

**Methods**

Imaging parameters

**Tables**

Additional file 1: Table S1. Cognitive functioning at baseline

Additional file 1: Table S2. Adjusted change in cognitive functioning in patients with normal variant versus foetal variant.

**Methods**

***Imaging parameters***

CTA was performed with a 16- or 64 row scanner (Philips Medical Systems, Best, the Netherlands) after injection of 50 mL contrast material at 5 mL/s, followed by a saline chaser bolus of 50 mL injected at the same flow rate. A 64x0.625 mm collimation was used, with a pitch of 0.672 and a rotation time of 0.40 seconds. Exposure settings were 80/120 kVp and 300/95 mAs. Overlapping sections of 1.0 mm (16^-^slice) or 0.9 mm slice thickness (40 or 64- slice) were reconstructed at a reconstruction interval of 0.5 mm and a field of view of 160 mm.

MRA was performed on 1.5 or 3.0 Tesla scanners (Philips Medical Systems, Best, the Netherlands) with a quadrature head coil for signal-intensity reception. The imaging protocol consisted of a 2D phase-contrast sagittal localizer survey through the circle of Willis, followed by a 3D-TOF MRA sequence with the following parameters: TR, 23 ms; TE, 3.5 ms; flip angle, 18°; sensitivity encoding factor, 2; FOV, 200 x 200 x 100 mm; matrix, 304 x 200 with 100 sections; reconstructed voxel size, 0.39 x 0.39 x 1.00 mm; and acquisition time, 2 minutes 57 seconds.

**Additional file 1:** **Table S1: Cognitive functioning at baseline**

| Test (n normal PCA, n foetal-type PCA) | Normal PCA | Foetal-type PCA |
| --- | --- | --- |
|  | (n=96) | (n=16) |
| Cognitive sum z-score* (96,16) | -0.19 (0.83) | -0.42 (0.72) |
| Baseline domain z-scores and raw test scores |  |  |
| Abstract Reasoning z-score (64,13) | -0.33 (0.79) | -0.62 (1.07) |
| Raven Advanced Progressive Matrices, short form (63,13) | 6.1 (2.5) | 4.9 (3.4) |
| WAIS similarities (64,13) | 18.8 (6.4) | 17.5 (7.6) |
| Attention z-score (95,16) | 0.68 (1.33) | 0.19 (1.24) |
| WAIS III Digit Span Forward (95,16) | 8.0 (2.2) | 6.9 (1.8) |
| Visual Elevator of the Test of Everyday Attention* (75,11) | 7.1 (2.8) | 6.1 (3.1) |
| Executive Functioning z-score (90,16) | -0.48 (0.70) | -0.73 (0.60) |
| Brixton Spatial Anticipation Task (86,16) | 20.9 (6.7) | 24.8 (5.9) |
| Letter Fluency† (87,16) | 9.2 (4.3) | 8.2 (3.5) |
| Language z-score (96,16) | -0.47 (1.11) | -0.67 (0.90) |
| Token Test, short form (89,16) | 16.4 (3.8) | 15.4 (3.9) |
| Boston Naming Test, short form (95,16) | 76.9 (12.5) | 75.9 (9.3) |
| Verbal Memory z-score (94,16) | -0.03 (1.15) | -0.17 (0.76) |
| WAIS III Digit Span backward (94,16) | 5.1 (2.2) | 4.6 (2.3) |
| Rey Auditory Verbal Learning Test‡ (93,16) | 19.8 (5.9) | 19.8 (5.0) |
| Semantic Fluency (86,16) | 26.3 (10.3) | 23.9 (5.7) |
| Visual Memory z-score (89,16) | -0.11 (0.90) | -0.16 (1.13) |
| Rey-Osterrieth Complex Figure-delay (89,16) | 15.5 (6.0) | 15.2 (7.6) |
| Visual Perception z-score (94,16) | -0.33 (0.93) | -0.70 (0.97) |
| Benton Judgment of Line Orientation, short form (88,15) | 22.6 (5.9) | 21.9 (7.3) |
| Facial Recognition Task, short form (89,16) | 43.2 (4.7) | 42.8 (3.8) |
| Rey-Osterrieth Complex Figure-copy (91,16) | 32.1 (5.0) | 29.0 (5.3) |
| Premorbid and current cognition |  |  |
| Informant Questionnaire of Cognitive Decline | 2.96 (0.36) | 3.02 (0.20) |
| Estimated premorbid Intelligent Quotient (NART) | 76.1 (21.4) | 71.2 (14.8) |
| MMSE | 26 [25-28] | 26 [24-27] |
| Premorbid anxiety/depression |  |  |
| STAI | 40.2 (12.5) | 38.9 (11.5) |
| STAT | 34.4 (9.8) | 30.2 (10.8) |
| BDI | 7.2 (6.5) | 6.2 (5.2) |
| Data are mean (SD) or median [range]. *Cognitive scores are expressed as units of standard deviations (z-scores) from the mean in a normal reference population, with negative values expressing scores below the normal population mean. *= mean of the visual elevator accuracy and timing score. †=mean of total words produced beginning with letter “N” or “A”. ‡= mean of the total direct, delayed, and recognized word count. CAS, carotid artery stenting; CEA, carotid endarterectomy; WAIS, Wechsler adult intelligence scale; NART, national adult reading test; MMSE, mini mental state examination; STAI, state-trait anxiety inventory (state); STAT, state trait anxiety inventory (trait); BDI, Beck’s depression inventory. | | |

| **Additional file 1:** **Table S2. Adjusted change in cognitive functioning in patients with normal variant versus foetal variant** | | |
| --- | --- | --- |
|  | Mean Difference | (95% CI) |
| Unadjusted cognitive sum z-score | -0.20 | (-0.40 to -0.01) |
| Additional adjustment |  |  |
| Age (years) | -0.20 | (-0.40 to -0.01) |
| Sex | -0.20 | (-0.40 to -0.01) |
| Education | -0.20 | (-0.39 to 0.00) |
| Side stenosis* | -0.22 | (-0.41 to -0.02) |
| Treatment | -0.20 | (-0.39 to -0.00) |
| Treated hypertension | -0.20 | (-0.40 to -0.00) |
| CABG | -0.20 | (-0.40 to -0.01) |
| Diabetes Mellitus | -0.20 | (-0.40 to -0.01) |
| Peripheral artery disease | -0.20 | (-0.39 to 0.00) |
| Smoker | -0.21 | (-0.40 to -0.01) |
| Degree symptomatic stenosis | -0.20 | (-0.40 to -0.01) |
| Contralateral degree of stenosis | -0.20 | (-0.39 to -0.01) |
| Presenting symptoms | -0.19 | (-0.39 to 0.01) |
| NIHSS | -0.20 | (-0.40 to -0.01) |
| Time interval* | -0.17 | (-0.38 to 0.03) |
| STAI* | -0.22 | (-0.41 to -0.03) |
| STAT | -0.21 | (-0.40 to -0.01) |
| BDI* | -0.22 | (-0.41 to -0.02) |
| 3 factors (age, sex, education) | -0.20 | (-0.40 to 0.00) |
| 4 factors ^*^ | -0.19 | (-0.38 to 0.01) |
| *Time interval represent the days between symptoms and treatment.  BDI indicates Beck’s depression inventory; CABG, coronary artery bypass grafting; CI, confidence interval; NIHSS, national institutes of health stroke scale; STAI, state-trait anxiety inventory (state); STAT, state trait anxiety inventory (trait). | | |
